# Self-organized traffic via priority rules in leaf-cutting ants

**DOI:** 10.1371/journal.pcbi.1006523

**Published:** 2018-10-11

**Authors:** Daniel Strömbom, Audrey Dussutour

**Affiliations:** 1 Department of Mathematics, Uppsala University, Uppsala, Sweden; 2 Department of Biosciences, Swansea University, Swansea, United Kingdom; 3 Department of Biology, Lafayette College, Easton, Pennsylvania, United States of America; 4 Centre de Recherches sur la Cognition Animale (CRCA), Centre de Biologie Intégrative (CBI), Université de Toulouse, CNRS, UPS, Toulouse, France; Santa Fe Institute, UNITED STATES

## Abstract

Ants, termites and humans often form well-organized and highly efficient trails between different locations. Yet the microscopic traffic rules responsible for this organization and efficiency are not fully understood. In previous experimental studies with leaf-cutting ants (*Atta colombica*), a set of local priority rules were isolated and it was proposed that these rules govern the temporal and spatial organization of the traffic on the trails. Here we introduce a model based on these priority rules to investigate whether they are sufficient to produce traffic similar to that observed in the experiments on both a narrow and a wider trail. We establish that the model is able to reproduce key characteristics of the traffic on the trails. In particular, we show that the proposed priority rules induce de-synchronization into clusters of inbound and outbound ants on a narrow trail, and that priority-type dependent segregated traffic emerges on a wider trail. Due to the generic nature of the proposed priority rules we speculate that they may be used to model traffic organization in a variety of other ant species.

## Author summary

Ants often form trails to transport food and supplies they find back to their nest. These trails have a function similar to the roads that connect people’s homes with the local mall, but while the traffic rules that cars on our roads are supposed to follow are well known the traffic rules ants use on their trails are still relatively unknown. Earlier experiments with leaf-cutting ants have suggested a set of simple traffic rules that ants may be attempting to follow on their trails. However, it is difficult to experimentally verify the link between the proposed rules and the observed traffic organization. Modeling is a useful way to link the behaviors isolated at the individual level and the pattern recorded at the collective level. Here we present and analyze a computational model based on the proposed traffic rules. We find that, with some modifications, the proposed rules are indeed sufficient to reproduce key features of the overall ant traffic observed in the experiments. Strengthening our belief that these traffic rules might be employed by the leaf-cutting ants to regulate traffic organization and due of their simplicity we speculate that similar rules may be used by other ant species.

## Introduction

Animal collective movement is a widespread phenomenon that occurs at various spatial and temporal scales in a variety of living organisms from cells to pedestrians [1–4]. Often there are no identifiable leaders or coordinators present and group coordination relies on a completely decentralized process. The global pattern is not explicitly encoded but emerges from numerous interactions between individuals that only have access to local and limited information [5–7].

In many situations the motion within the collective is unidirectional because it is related to migratory phenomena and involve individuals moving in the same direction. Social insects and humans are some of the rare organisms in which the movements within the collective are predominantly bidirectional [8–12]. In particular, ants are central-place foragers and must return to their nest with the food collected after each foraging event which often lead to the formation of trails with a steady stream of traffic between the nest and the food source. In some species the traffic flow on these trails can be extremely high, reaching more than a hundred ants per minute, e.g. red wood ants [13], leaf cutting ants [14] and army ants [15]. When the local concentration of individuals is very high on the trail the high rate of head-on collisions may slow down the individuals [16–18]. These effects can provoke group dysfunctions and reduce the colony’s overall foraging efficiency. Such negative effects can be avoided if ants make use of dispersal mechanisms allowing a better organization of the traffic [8].

Traffic in ants can be organized either on a spatial or on a temporal scale [8]. Spatial organization of traffic is characterized by lane segregation, i.e. the flows of inbound and outbound ants are not completely intermingled [19–21] and the temporal organization of the flow is characterized by a sequence of alternating clusters of inbound and outbound ants [16,22]. To date, the emergence of both the spatial and the temporal organizations observed in these systems remains largely unexplained. In particular, the microscopic traffic rules that individual ants follow when navigating on trails are largely unknown.

In an attempt to isolate the microscopic traffic rules experimental studies on traffic organization in leaf-cutting ants *Atta colombica* on a narrow trail [22] and on a wide trail [20] were performed. Leaf-cutting ant trails guide workers to and from the foraging site where they cut vegetation into small fragments and transport them back to the nest. These fragments are then incorporated into a fungus on which the colony feed. In the experiments, to reach the leaf source, ants were forced to move on either a narrow trail allowing the passage of only one moving individual at a time [22] or on a wide trail ten times larger [20]. On the narrow trail de-synchronization of inbound and outbound traffic involving the formation of alternating clusters of inbound and outbound ants was observed and on the wide trail a degree of lane segregation with leaf carrying ants travelling almost exclusively on the central section of the trail was described. Summaries of the results obtained in the experiments may be found in the supporting information S1 Table (narrow trail) and S2 Table (wide trail). The authors suggested that both organizations may result from a set of local priority rules observed at the individual level when ants encountered other ants on the trail [20, 22]. However, whether these proposed individual priority rules are sufficient to produce the observed temporal and spatial traffic organization is unknown and to investigate this modelling is required.

There are many well known models for ant traffic, most of which are mean-field models studying the macroscopic properties of the traffic on trails [23–31]. However, to investigate the group level traffic that emerges from repeated local interactions between moving individuals so-called self-propelled particle models are more appropriate. Self-propelled particle models are spatially-explicit individual based models where particles interact locally with each other according to a set of rules. These models range from minimal models used to investigate fundamental properties of collective motion [32–35] to more involved species specific models of collective motion in everything from cells to insects, fish, birds, sheepdogs and pedestrians [9,10,36–44]. The self-propelled particle model approach has been successfully applied to model ant traffic in army ants [19] and black garden ants [45]. However, in these models the characteristic features of outbound and nest-bound ants were not distinguished despite the fact that variation in their maneuverability and speeds exist due to food transport [22, 46].

Here we introduce a self-propelled particle model to reproduce the experiments with leaf-cutting ants *Atta colombica* on a narrow trail [22] and on a wide trail [20], and to investigate whether the local priority rules proposed in these studies are sufficient to reproduce the traffic organization observed.

## Models and methods

### Model for the narrow trail

To focus our investigation on which macroscopic properties of the traffic the local priority rules alone are responsible for we simplify the model ants (particles) in several ways. In particular, particles are assumed to have constant length 1cm and constant type dependent speeds. Mimicking the experimental setup the particles move on a 300 cm long one-dimensional trail connecting the nest and a leaf source. Particles leave the nest and the leaf source according to a Poisson process characterized by the rate parameter μ. At the beginning of each simulation particles are present at the nest and the leaf source. Following the experimental work we consider three types of particles: outbound (O), inbound unladen (U) and laden particles (L). The probability of a particle being laden when leaving the leaf source is 0.24 as observed in the experiment (S1 Table). Due to the small difference in speed observed between outbound (O) and unladen ants (U) we set the speed to *s*_*OU*_ = 2.3 cm/s for both types of particles. The speed of laden particles (L) is set to *s*_*L*_ = 1.9 cm/s (S1 Table).

On the narrow trail the positional update formula for each particle is given by

x(t+Δt)=x(t)+δ(t)shΔt,

where *x*(*t*) is the *x*-coordinate of the particle on the trail at time *t* (the nest is at *x* = 0 and the leaf source at *x* = 300), *s* ∈ {*s*_*OU*_, *s*_*L*_} is the speed of the particle, *h* ∈ {−1, 1} is the heading of the particle (1 for outbound and -1 for inbound particles), and Δ*t* = 0.1 is the time step. The value of *δ*(*t*) ∈ {0, 1} depends on the interactions between particles at time *t*. *δ*(*t*) is 1 if the particle is given the way, and 0 if the particle stops and gives the way. Two particles interact when they are within a distance of 1 + 2 Δ*t s*_*OU*_ of each other to account for the size of the particles. The interactions between particles are specified by a set of local priority rules identified in [22];

1. An unladen particle (U) does not attempt to pass a laden particle (L) ahead of it. Instead it stops and waits until the laden ant has moved forward enough for it to take another step.
2. An outbound particle (O) stops and gives way to a laden particle (L), and potentially a number of unladen particles (U) following the laden particle (See The cooperative rule below).
3. An unladen particle (U) stops and gives way to an outbound particle (O), unless the outbound particle (O) is waiting following an interaction with a laden particle (See The cooperative rule below).

In order to cover all interaction possibilities we also included the following two rules not quantified in [22];

4. A particle of any type (O, L and U) does not attempt to pass another particle of the same type ahead of it. Instead it stops and waits until the particle has moved forward enough for it to take another step.
5. A laden particle (L) does not stop for stationary inbound unladen particles (U) ahead of it.

#### The cooperative rule

When an outbound particle gives way to a laden particle a number (*n* = 0,1, …,15) of unladen particles traveling in line behind the laden ant may also be allowed passage. This phenomenon was referred to as “the cooperative rule” in [22] and the probability that *n* unladen ants benefited from the passage of a laden ant in this way in the experiments was quantified and presented in (Fig 6 in [22]). Here we use the experimentally observed probabilities and implement the cooperative rule as follows. Based on particle speed, an unladen particle takes approximately one second to pass the stationary outbound particle directly after the laden particle has passed and the experimentally determined time loss per contact was 0.8 seconds (S1 Table). So in order to let *n* unladen particles pass the outbound particle (O) waits for *τ* = 0.8*n* seconds after encountering the laden particle before it starts to move again. Once the outbound particle starts to move it has priority over unladen particles again. The traffic rules for particles on the narrow trail are summarized in Table 1.

**Table 1:** Particle traffic rules on the narrow trail.

	O Behavior	U Behavior	L Behavior
When encountering O	STOP	STOP*	WALK
When encountering U	WALK*	STOP	WALK
When encountering L	STOP	STOP	STOP

The asterisk (*) indicates potential exceptions due to the cooperative rule (See description in the main text).

There is one unknown parameter in the model: the rate parameter *μ* associated with the Poisson process dictating the flow of particles leaving the nest and the leaf source. We know from the experiments [22] that the total number of ants crossing the middle of the trail in one hour on average was 5418.8 particles (S1 Table). Using simulations we found that the generic value μ = 1 produced a flow of 5347.2 (s.d. 73.5) over 1000 simulations, so we used this when running simulations to compare with the experiments.

### Model for the wide trail

**Fig 1:**
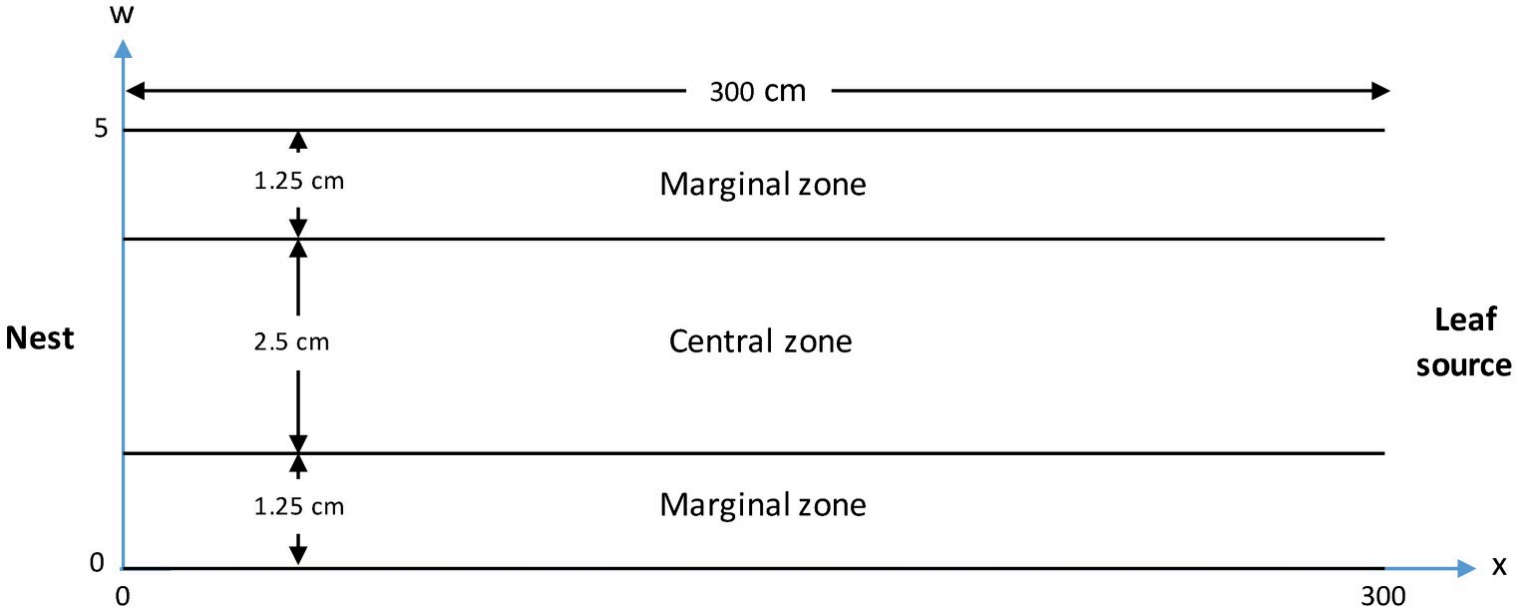
Schematic of the wide trail. Illustrating the definitions of the central and marginal zones and the (*x*,*w*)-coordinate system used in the model.

The wide trail experiment [20] was conducted using the same experimental procedure as in the narrow trail experiment and the results are summarized in S2 Table. Ants were forced to move on a 5cm wide and 300cm long trail linking the nest and the leaf source (Fig 1). In addition to the observations made in the narrow trail experiments on the wide trail the spatial organization of the traffic was also quantified. The number and type of ants (O, U and L) passing the midpoint of the trail in three different zones; a central zone (2.5cm wide) and two marginal zones (each 1.25cm wide) were recorded (Fig 1).

The main difference between the narrow and wide trail settings is that there is room for ants/particles to turn on the wide trail so they are not forced to stop when encountering an ant/particle with higher priority. Instead the ant/particle can turn to avoid collision and we adapted the narrow trail model to the wide trail by replacing all instances of stop with turn. The traffic rules for particles on the wide trail are presented in Table 2.

**Table 2:** Particle traffic rules on the wide trail.

	O Behavior	U Behavior	L Behavior
When encountering O	TURN	TURN	WALK
When encountering U	WALK	TURN	WALK
When encountering L	TURN	TURN	TURN

Same as the narrow trail rules (Table 1) with all instances of Stop replaced by Turn and the cooperative rule removed.

The positional update formula for each particle in the wide trail model is

$$\{\begin{array}{c} x\left(t+\Delta t\right)=x\left(t\right)+s\;cos\left(\theta \left(t\right)\right)\Delta t\\ w\left(t+\Delta t\right)=w\left(t\right)+s\;sin\left(\theta \left(t\right)\right)\Delta t \end{array}$$

where *x*(*t*) is the *x*-coordinate of the particle on the trail at time *t* (the nest is at *x* = 0 and the leaf source at *x* = 300), *w*(*t*) is the *w*-coordinate of the particle at time *t* (the walls are located at *w* = 0 and *w* = 5), *s* ∈ {*s*_*OU*_, *s*_*L*_} is the speed of the particle, Δ*t* = 0.1 is the time step, and *θ(t)* is the heading of the particle at time *t*. The heading *θ*(*t*) = *θ*_*d*_ +*θ*_*I*_(*t*) is composed of two components:*θ*_*d*_ ∈ {0, *π*} which is equal to 0 for outbound and *π* for inbound particles, and *θ*_*I*_ ∈ {−*π*/2,0,*π*/2} which is the turning angle resulting from an interaction and depends on the traffic rules (Table 2). During an interaction a particle *i* will not turn (*θ*_*I*_ = 0) if it is given the way by particle *j*. It will turn if it is not given the way by particle *j*. In that latter case, it will turn up if *w*_*i*_ (*t*) *≥ w*_*j*_ (*t*) or down if *w*_*i*_ (*t*) < *w*_*j*_ (*t*). Note that when a particle turns its displacement in the x-direction is 0, because $cos(\pm \frac{\pi }{2})\;=\;0$, so its arrival to the nest/leaf site is delayed by Δ*t* = 0.1s by each turn time step.

The cooperative rule where unladen following a laden are sometimes allowed passage is not relevant in the wide trail setting because particles are turning instead of stopping. As in the narrow trail case we use the experimentally observed total flow to set the rate parameter *μ* associated with the Poisson process of ants leaving the nest and leaf source. The average flow in the wide trail experiments was 8803 ants/hour and choosing *μ* = 0.8 results in a flow of 8999.4 (s.d. 97.8) and we use this value for the simulations. Unlike in the narrow trail case, where all ants entered and moved on the trail at the same vertical position, on the wide trail ants entered the trail from the nest via a small opening centered on the trail and from the leaf source via a narrow wooden stick attached to the center of the trail. So the ants entered close to the center on both sides of the trail and then scattered in the w-direction. To model this we assume that the vertical entry position (*w*(0)) for each ant is normally distributed with mean 2.5 (center of the trail) and standard deviation 0.8. This ensures that almost all entry positions lie between 0 and 5 and if the generated *w*(0)>5 we set *w*(0) = 4.99 and if *w*(*0*)<0 we set *w*(0) = 0.01.

To analyze the model we ran 1000 simulations and calculated the proportion of each particle type in each zone of the trail, in addition to the mean and maximum group sizes, and compared the results with the experimental results (S2 Table). We also ran a set of simulations with turning replaced by stopping as on the narrow trail to investigate the causal effects of turning with respect to the spatial organization on the wide bridge.

## Results

### Comparison model-experiment for the narrow trail

**Fig 2:**
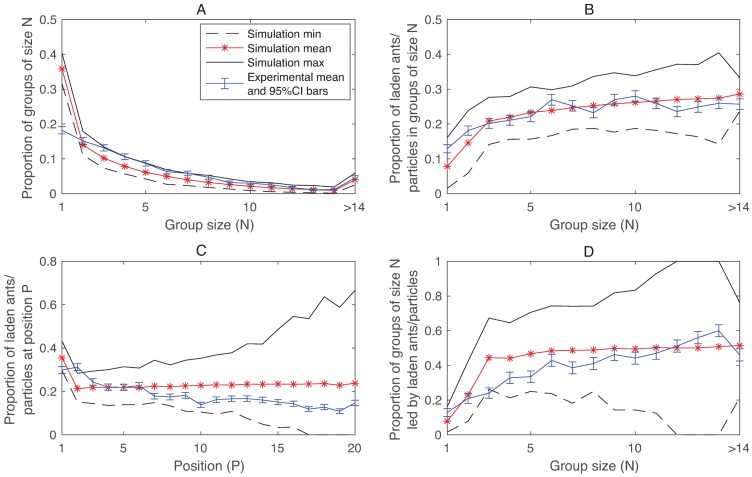
Comparison model-experiment for the narrow trail. In each plot the blue curves represent the experimental means and the values correspond to those presented in Figs 2–5 in [22]. The red asterisks represent the simulation means and the black lines represent the maximum and the minimum over 1000 simulations.

The main experimental finding was that a de-synchronization of inbound and outbound traffic occurred on the narrow trail that involved the formation of alternating groups of inbound and outbound ants. De-synchronization of this type also emerged in simulations of the model and the resulting groups share several properties with the experimentally observed groups. See S1 Fig for an illustration of the concept of de-synchronization. In [22] four statistics were used to quantify the traffic organization and structure of the groups: (i) group size distribution, (ii) proportion of laden ants in groups of size *N*, (iii) proportion of laden ants at position *P* in a group, and (iv) proportion of groups of size *N* led by a laden ant. A comparison of the experimentally observed distributions and the distributions generated from simulations are presented in Fig 2. We see that overall the distributions generated by simulations are comparable with those obtained in the experiments. In particular, for group sizes larger than 1 the experimental means are essentially contained within the min-max bounds of the distributions generated by simulations for all four statistics. The main discrepancy between simulations and experiments is the overrepresentation of groups of size 1 in the group size distribution generated by simulations (Fig 2A). In addition, the maximum group size observed in experiments was *74* (S1 Table) and in simulations *77*, and the average group size observed in experiments was *5*.*2* (S1 Table) and in simulations *4*.*2*.

### Comparison model-experiment for the wide trail

**Fig 3:**
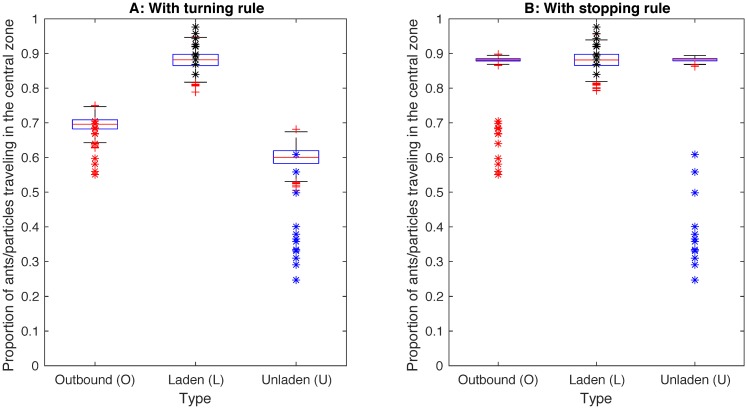
Comparison model-experiment for the wide trail. The asterisks represent the proportion of ants of the specific type traveling in the central zone on the trail in the experiments. The boxes represent corresponding measurements over 1000 simulations (red crosses represents outliers) of (A) The wide trail model with the turning rule, and (B) The wide trail model with the stopping rule instead of the turning rule.

The main finding in the experiment was that a degree of lane segregation occurred on the wide trail. Laden ants returning to the nest travelled mostly in the central zone of the trail, while unladen ants (U) more frequently travelled in the marginal zones of the trail. For outbound ants (O) about two thirds of them travelled in the central zone of the trail. The asterisks in Fig 3A and 3B shows the results for each type of ants in each of the 12 experiments. The boxplots represent the corresponding measurements in simulations with the wide bridge model using the turning rule (Fig 3A) or the stopping rule (Fig 3B). We found that the wide bridge model using the turning rule reproduces the overall trend of laden particles moving almost exclusively in the central zone and outbound particles travelling in the central zone more frequently than inbound unladen particles (Fig 3A). We also found that in the simulations the average group size was 2.0 and the maximum group size 23, compared to the experimental average group size of 1.9 and maximum group size 14 (S2 Table). In addition, Fig 3B shows that when the turning rule is replaced by a stopping rule, lane segregation does not emerge and we conclude that lane segregation is critically dependent on turning in our model.

## Discussion

Our self-propelled particle model based on the local priority rules presented in [22] generates traffic organization that share several characteristics with the traffic organization observed in the narrow and wide trail experiments. In particular, the narrow trail model reproduces de-synchronization of inbound and outbound traffic and the groups that emerge share several features with the experimentally observed groups (Fig 2). The wide trail model generated segregated traffic that share certain properties with the traffic observed in the experiments (Fig 3). In particular, that the proportion of ants of a given type (L, O or U) traveling in the central zone increased with the priority of each type (L>O>U) in both model and experiment.

This suggests that it is plausible that the priority rules proposed in [22] are key drivers of organizing the traffic on these trails because the main features of the observed traffic emerge from them even when the rest of the system is heavily idealized. For example, we use simplified model ants with constant speed and lengths that always follow the rules and the only stochastic components in the models are related to leaving times and entry positions. In particular, we believe that the strict rule following produces the main discrepancy between simulation results and data, i.e. the over representation of groups of size 1 on the narrow trail (Fig 2A). We also know that introducing various types of stochastic rule violations in the model does not solve this problem, and we are confident that the rule violations are far from randomly occurring and new experiments would be required to investigate this. However, while it would be interesting and potentially useful to conduct new experiments to obtain data that allows us to make certain aspects of the model more realistic this would inevitably make the model more complex and thus make it harder to isolate the effects of generic mechanisms underlying traffic organization.

Understanding the basic principles that govern traffic organization on trails and identifying the factors that influence the movements of ants on trails are of fundamental importance in the biology of social insect colonies. When ants were forced to move on a narrow trail the formation of alternating groups of inbound and outbound ants was observed. Groups of inbound ants were frequently headed by laden ants, which are slower, followed by unladen ants. The model replicates this behavior, most likely due to the rule that dictates that inbound unladen ants do not attempt to overtake laden ants in front of them (Rule 1). This behavior may appear detrimental because unladen ants move slower by staying behind a laden ant instead of progressing more rapidly by moving at its desired speed. However, the model also includes the so-called cooperative rule that allows for the possibility of unladen ants following a laden ant to benefit from the passage of the laden (See rules 2 and 3). These unladen ants avoid head-on collisions with outbound ants and thus spare time they would otherwise waste by stopping as they normally do when they meet an outbound ant. Moreover, this organization promotes information transfer about the level of leaf availability by increasing the number of contacts between outbound and inbound laden ants which stimulate the former to cut and retrieve leaf fragments when reaching the end of the trail [47–53]. Following the same idea, on the wide trail, an intermingled flow of outbound and unladen ants instead of a strict lane segregation might appear sub-optimal, but it actually promotes information transfer between ants and stimulate outbound workers to cut and collect leaf material at the end of the trail, thus contributing to increased foraging efficiency [8, 20, 48, 49].

Our findings suggest that the ants may be using the same generic priority rules on both trails and the observed differences results from constraints imposed by the environment. In particular, on a wider trail there is room to turn during an encounter whereas on a narrow trail this option is not available so the ants have to stop when giving way. In fact, due to the simplicity of these rules we believe that they could be valid, with appropriate modifications, for other species of ant under similar conditions. For example, the same priority rule between laden and unladen ants has been observed in another leaf-cutting ant *Atta cephalotes* [47] and in the red wood ant *Formica rufa* [13]. In addition, similar types of priority rules are likely to be operating in army ants because returning laden ants are known to be less mobile and have less maneuverability than unladen outbound ants [46]. We also note that there is a correspondence between the priority rules and the potential utility of each type of ant with respect to leaf collection. Laden ants have the highest priority and they *are* collecting leaves, outbound ants have the second highest priority and they *are potentially* going to collect leaves, and inbound unladen ants have the lowest priority and they *are not* collecting leaves. We speculate that priority rules in other species are likely to correspond to the potential utility of each type of ant with respect to the colony’s foraging activity.

Our model distinguishes itself from earlier spp-models of ant traffic in several ways. In particular, we model three types of ants (outbound, unladen and laden) whereas [19,45] only include two; inbound and outbound, and the two types are essentially identical except for different avoidance turning rates in [19]. Furthermore, the avoidance turning rate is the same for all individuals of a certain type, i.e. outbound or inbound, despite the fact that variation in maneuverability and speeds exist in real ants due to food transport [22, 46]. Our model includes this variability and our priority rules are flexible enough to model traffic on both narrow and wide trails. In [19,45] only traffic on wider trails are modeled and while the avoidance turning rate approach may be modified to work on narrow trails, which presumably both army ants [19] and black garden ants [45] occasionally travel on in the wild, we predict that unless the inbound flow is separated into unladen and laden ants with different behaviors the model will not be able to generate traffic consistent with the real ant traffic [16].

One often thinks about the similarities between ant traffic, pedestrian traffic and vehicular traffic. These analogies have inspired multiple investigations [54–58]. However, even if at first sight traffic on ant trails may appear similar to human traffic there are important differences to consider when comparing their traffic organization. First, ant traffic is of a cooperative nature because all ants share a common objective, namely harvesting food for the colony. Second, ants do not have the same mechanical constraints as pedestrians or vehicles. Because of their small mass they have a low inertia and are not damaged by collisions, allowing a certain degree of mixing of opposite flows on foraging trails. Despite this, ant traffic remains an important source of inspiration for various researchers working with large groups of interacting particles in disciplines as diverse as molecular biology [59], statistical physics [60] and telecommunication sciences [61].

## Supporting information

S1 Table Summary of the narrow trail experiment and results. From [22] based on 12 replicates. The trail linking the nest to the food source was 0.5cm wide and 300cm long. The flow of ants leaving the nest (outbound ants) and that leaving the leaf source (inbound ants) was counted in 1 min intervals for 1h and the proportion of laden ants in the inbound flow was measured. The formation of groups of successive ants travelling in the same direction in the sequence of ants observed on the trail was quantified. At the individual level, the ants speed and the outcome of head-on collisions between ants (priority rule) were measured. Four types of ants were distinguished: outbound ants (O), (inbound) unladen ants (U), (inbound) laden ants (L), and unladen ants following a laden ant (U(L)). The outcome of head-on collision between outbound ants and laden ants (O vs L), outbound ants and unladen ants (O vs U), and unladen ants and laden ants (U vs L) was analyzed. Typically, after a collision one ant moves to the trail side (STOP) to allow the passage of the oncoming ant (WALK). The time loss per collision was measured and it did not differ according to the type of ants involved. When an ant gave way to another, it generally moved to the side of the trail and allowed the passage of the ant. The follower ants might benefit from the passage of the leading ant (the ant that was given way) before the ant that gave way returns to the top of the trail. This latter effect corresponds to a cooperative behavior between ants because the subsequent ants benefit from the passage of the leading ant. The probability of an ant to benefit from the passage of the leading ant depended both on its position as a follower and on the category of the leading ant (O, U or L). *N* indicates the number of observation used to obtain the mean value or rule.
(DOCX)

S2 Table Summary of the wide trail experiment and results. From [20] based on 12 replicates. The trail linking the nest to the food source was 5cm wide and 300cm long. The flow of ants leaving the nest (outbound ants) and that leaving the food source (inbound ants) was counted in 1 min intervals for 1h and the proportion of laden ants in the inbound flow was measured. The formation of groups of successive ants travelling in the same direction in the sequence of ants observed on the trail was quantified. At the individual level, the ants speed and the outcome of head-on collisions between ants (priority rule) were measured. Three types of ants were distinguished: outbound ants (O), unladen (U) ants and laden ants (L). The outcome of head-on collisions between outbound ants and inbound laden ants (O vs L), and outbound ants and inbound unladen ants (O vs U) were analyzed. Typically, after a collision occurred one ant turns (TURN) to allow the passage of the oncoming ant (WALK). N indicates the number of observation used to obtain the mean value or rule.
(DOCX)

S1 Fig Illustrating the concept of de-synchronization of inbound and outbound traffic. At time 0 there are outbound particles (red dots), inbound unladen particles (blue dots), and an inbound laden particle (black dot) near the middle of the trail. Over time the red particles will move towards the leaf source (right) and the black and blue particles towards the nest (left), and when a particle crosses the middle of the trail we record a crossing as +1 if it is outbound, -1 if inbound unladen, and -2 if inbound laden. We note that on time steps 1 and 2 we record inbound crossings (-1). On time step 3 the first interaction where one unladen particle steps off the trail in order to give way to an outbound ant. This process continues according to the model rules and by the time step 14 we have collected the sequence -1,-1,+1,+1,+1,-2,-1,-1,-1 and from this we calculate group size by counting consecutive entries with the same sign. Here we first had a group of 2 inbound particles (-1,-1), then a group of 3 outbound particles (+1,+1,+1), and then a group of 4 inbound particles (-2,-1,-1,-1). This phenomenon is referred to as a de-synchronization of inbound and outbound traffic involving the formation of alternating groups of inbound and outbound ants in [22] and we use the same terminology for particles here.
(TIF)

## References

[1] Sumpter D. Collective animal behavior. Princeton University Press; 2010.

[2] Vicsek T, Zaferidis A. Collective motion. Phys Rep. 2012; 517: 71–140.

[3] Parrish J, Hamner W. Animal Groups in Three Dimensions. Camtrail University Press; 1997.

[4] Heppner F. Avian flight formations. Bird-Banding. 1974; 45: 160–169.

[5] Camazine S, Deneubourg JL, Franks N, Sneyd J, Theraulaz G, et al Self-Organization in Biological Systems. Princeton University Press; 2001.

[6] Parrish J, Viscido S, Grünbaum D. Self-organized fish schools: an examination of emergent properties. J Comp Physiol. 2001; 135: 315–325. 10.2307/1543482 12087003

[7] Couzin I, Krause J. Self-organization and collective behavior in vertebrates. Adv Stud Behav. 2003; 32: 1–75.

[8] Fourcassié V, Dussutour A, Deneubourg JL. Ant traffic rules. J Exp Biol. 2010; 213: 2357–2363. 10.1242/jeb.031237 20581264

[9] Helbing D, Molnr P, Farkas I, Bolay K. Self-organizing pedestrian movement. Environ Plann B. 2001; 28: 361–383.

[10] Moussaïd M, Helbing D, Theraulaz G. How simple rules determine pedestrian behavior and crowd disasters. Proc Natl Acad Sci USA. 2011; 108: 6884–6888. 10.1073/pnas.1016507108 21502518 PMC3084058

[11] Moussaïd M, Guillot E, Moreau M, Fehrenbach J, Chabiron O, et al Traffic instabilities in self-organized pedestrian crowds. PLoS Comput Biol. 2012; 8(3): e1002442 10.1371/journal.pcbi.1002442 22457615 PMC3310728

[12] John A, Schadschneider A, Chowdhury D, Nishinari K. Collective effects in traffic on bi- directional ant trails. J Theor Biol. 2004; 231: 279–285. 10.1016/j.jtbi.2004.06.022 15380392

[13] Holt S. On the foraging activity of the wood ant. J Anim Ecol. 1955; 24: 1–34.

[14] Wirth R, Herz H, Ryel R, Beyschlag W, Hölldobler B. Herbivory of leaf-cutting ants: a case study on *Atta colombica* in the tropical rainforest of Panama. Springer Science & Business Media; 2013.

[15] Gotwald WH. Army ants: the biology of social predation. Cornell University Press; 1995.

[16] Dussutour A, Deneubourg JL, Fourcassié V. Temporal organization of bi-directional traffic in the ant *Lasius niger*. J Exp Biol. 2005; 208: 2903–2912. 10.1242/jeb.01711 16043595

[17] Dussutour A, Nicolis S, Deneubourg JL, Fourcassié V. Collective decisions in ants when foraging under crowded conditions. Behav Ecol Sociobiol. 2006; 61: 17–30.

[18] Burd M, Aranwela N. Head-on encounter rates and walking speed of foragers in leaf-cutting ant traffic. Insectes Soc. 2003; 50: 3–8.

[19] Couzin I, Franks N. Self-organized lane formation and optimized traffic flow in army ants. Proc R Soc Lond B. 2003; 270: 139–146. 10.1098/rspb.2002.2210 PMC1691225 12590751

[20] Dussutour A. Organisation spatio-temporelle des déplacements collectifs chez les fourmis. Doctoral dissertation, Toulouse 3; 2004

[21] Hönicke C., Bliss P., & Moritz R. F. (2015). Effect of density on traffic and velocity on trunk trails of Formica pratensis. The Science of Nature, 102(3–4), 17 10.1007/s00114-015-1267-6 25813053

[22] Dussutour A, Beshers S, Deneubourg JL, Fourcassie V. Priority rules govern the organization of traffic on foraging trails in the leaf-cutting ant *Atta colombica*. J Exp Biol. 2009; 212: 499–505. 10.1242/jeb.022988 19181897

[23] Deneubourg JL, Goss S, Franks N, Pasteels J. The blind leading the blind: modelling chemically mediated army ant raid patterns. J Insect Behav. 1989; 2: 719–725.

[24] Stickland T, Britton N, Franks N. Algorithms for ant foraging. Naturwissenschaften. 1993; 80: 427–430.

[25] Stickland T, Britton N, Franks N. Complex trails and simple algorithms in ant foraging. Proc R Soc B. 1995; 260: 53–58.

[26] Britton N, Stickland T, Franks N (1998) Analysis of ant foraging algorithms. J Biol Syst 6: 315–336.

[27] Nicolis S, Deneubourg JL. Emerging patterns and food recruitment in ants: an analytical study. J Theor Biol. 1999; 198: 575–592. 10.1006/jtbi.1999.0934 10373356

[28] Beekman M, Sumpter D, Ratnieks F. A phase transition between disordered and ordered foraging in pharoahs ants. Proc Natl Acad Sci USA. 2001; 98: 9703–9706. 11493681 10.1073/pnas.161285298 PMC55516

[29] Dussutour A, Fourcassié V, Helbing D, Deneubourg JL. Optimal traffic organization in ants under crowded conditions. Nature. 2004; 428: 70–73. 10.1038/nature02345 14999281

[30] Johnson K, Rossi L. A mathematical and experimental study of ant foraging trail dynamics. J theor Biol. 2006; 241: 360–369. 10.1016/j.jtbi.2005.12.003 16442564

[31] John A, Schadschneider A, Chowdhury D, Nishinari K. Trafficlike collective movement of ants on trails: Absence of a jammed phase. Phys Rev Let. 2009; 102: 108001 19392163 10.1103/PhysRevLett.102.108001

[32] Vicsek T, Czirók A, Ben-Jacob E, Cohen I, Shochet O. (1995) Novel type of phase transition in a system of self-driven particles. Phys Rev Let. 1995; 7: 1226–1229. 10.1103/PhysRevLett.75.1226 10060237

[33] Strömbom D. Collective motion from local attraction. J Theor Biol. 2011; 283: 145–151. 10.1016/j.jtbi.2011.05.019 21620861

[34] Romanczuk P, Couzin I, Schimansky-Geier L. Collective motion due to individual escape and pursuit response. Phys Rev Let. 2009; 102: 010602. 19257176 10.1103/PhysRevLett.102.010602

[35] Strömbom D, Siljestam M, Park J, Sumpter D. The shape and dynamics of local attraction. Eur Phys J ST. 2015; 224: 17–18.

[36] Aoki I. A simulation study on the schooling mechanism in fish. Bull Japan Soc Sci Fish. 1982; 48: 1081–1088

[37] Huth A, Wissel C. The simulation of the movement of fish schools. J Theor Biol. 1991; 156: 365–385.

[38] Couzin I, Krause J, James R, Ruxton G, Franks N. Collective memory and spatial sorting in animal groups. J Theor Biol. 2002; 218: 1–11. 12297066 10.1006/jtbi.2002.3065

[39] Isobe M, Helbing D, Nagatani T. Experiment, theory, and simulation of the evacuation of a room without visibility. Phys Rev E. 2004; 68: 6884–6888. 10.1103/PhysRevE.69.066132 15244692

[40] Hemelrijk C, Kunz H. Density distribution and size sorting in fish schools: an individual- based model. Behav Ecol. 2005; 16: 178–187.

[41] Parrish J, Viscido S. Traffic rules of fish schools: a review of agent-based approaches In: Hemelrijk C Editor. Self-organisation and the evolution of social behaviour; 2005.

[42] Hildenbrandt H, Carere C, Hemelrijk C. Self-organized aerial displays of thousands of starlings: a model. Behav Ecol. 2010; 21: 1349–1359.

[43] Lukeman R, Li X, Edelstein-Keshet L. Inferring individual rules from collective behaviour. Proc Natl Acad Sci USA. 2010; 107: 12576–80. 10.1073/pnas.1001763107 20616032 PMC2906562

[44] Strömbom D, Mann R, Wilson A, Hailes S, Morton A, et al Solving the shepherding problem: Heuristics for herding autonomous, locally interacting agents. JR Soc Interface 2014; 11 100: 20140719. 10.1098/rsif.2014.0719 PMC4191104 25165603

[45] Koutsou A, He S. Study of ants traffic organisation under crowded conditions using individual-based modelling and evolutionary computation. IEEE Trans Evol Comput. 2009; 2: 25–41.

[46] Zollikofer C. Stepping patterns in ants—influence of body morphology. J Exp Biol. 1994; 192: 107–118. 9317436 10.1242/jeb.192.1.107

[47] Burd M, Archer D, Aranwela N, Stradling D. Traffic dynamics of the leaf-cutting and, *Atta cephalotes*. Am Nat. 2002; 159: 283–293. 10.1086/338541 18707380

[48] Dussutour A, Beshers S, Deneubourg JL, Fourcassié V. Crowding increases foraging efficiency in the leaf-cutting ant *Atta colombica*. Insectes Soc. 2007; 54: 158–165.

[49] Dussutour A., Deneubourg J. L., Beshers S., & Fourcassié V. (2009). Individual and collective problem-solving in a foraging context in the leaf-cutting ant Atta colombica. Animal cognition, 12(1), 21. 18560906 10.1007/s10071-008-0165-0

[50] Farji-Brener A, Chinchilla F, Rifkin S, Cuervo AS, Triana E, et al The ‘truck-driver effect in leaf-cutting ants: how individual load influences the walking speed of nest-mates. Physiol Entomol. 2011; 36: 128–134.

[51] Roces F, Bollazzi M. Information transfer and the organization of foraging in grass- and leaf-cutting ants In: Jarau S, Hrncir M Editors. Food Exploitation by Social Insects: Ecological, Behavioral, and Theoretical Approaches. CRC Press; 1999.

[52] Bouchebti S., Ferrere S., Vittori K., Latil G., Dussutour A., & Fourcassié V. (2015). Contact rate modulates foraging efficiency in leaf cutting ants. Scientific reports, 5, 18650 10.1038/srep18650 26686557 PMC4685442

[53] Cibils-Martina L., Elizalde L., & Farji-Brener A. G. Traffic rules around the corner: walking of leaf-cutting ants at branching points in trunk trails. Insectes Sociaux, 1–7.

[54] Nishinari K, Sugawara K, Kazama T, Schadschneider A, Chowdhury D. Modelling of self-driven particles: Foraging ants and pedestrians. Physica A. 2006; 372:132–141.

[55] Parisi DR, Soria SA, Josens R. Faster-is-slower effect in escaping ants revisited: Ants do not behave like humans. Safety Sci. 2015; 72, 274–282.

[56] Gottlieb J, Puchta M, Solnon C. A study of greedy, local search, and ant colony optimization approaches for car sequencing problems In: Springer Berlin Heidelberg Applications of evolutionary computing (.; 2004; 246–257.

[57] Schadschneider A, Kirchner A, Nishinari K. From ant trails to pedestrian dynamics. Appl Bionics Biomech. 2003; 1, 11–19.

[58] John A, Schadschneider A, Chowdhury D, Nishinari K. Characteristics of ant-inspired traffic flow. Swarm Intell. 2008; 2, 25–41.

[59] Tabony J. Microtubules viewed as molecular ant colonies. Biol Cell. 2006; 98: 603–617. 10.1042/BC20050087 16968217

[60] Chowdhury D, Nishinari K, Schadschneider A. Self-organized patterns and traffic flow in colonies of organisms: from bacteria and social insects to vertebrates. Phase Transit. 2004; 77: 601–624.

[61] Blum C. Ant colony optimization: introduction and recent trends. Phys Life Rev. 2005; 2: 353–373.

