# Supplementary material for: Self-organized traffic via priority rules in leaf-cutting ants
Source: PLoS Comput Biol. 2018 Oct 11;14(10):e1006523. doi: 10.1371/journal.pcbi.1006523 (PMC6198993; doi:10.1371/journal.pcbi.1006523)
Supplement: S1 Table — From [22] based on 12 replicates. The trail linking the nest to the food source was 0.5cm wide and 300cm long. The flow of ants leaving the nest (outbound ants) and that leaving the leaf source (inbound ants) was counted in 1 min intervals for 1h and the proportion of laden ants in the inbound flow was measured. The formation of groups of successive ants travelling in the same direction in the sequence of ants observed on the trail was quantified. At the individual level, the ants speed and the outcome of head-on collisions between ants (priority rule) were measured. Four types of ants were distinguished: outbound ants (O), (inbound) unladen ants (U), (inbound) laden ants (L), and unladen ants following a laden ant (U(L)). The outcome of head-on collision between outbound ants and laden ants (O vs L), outbound ants and unladen ants (O vs U), and unladen ants and laden ants (U vs L) was analyzed. Typically, after a collision one ant moves to the trail side (STOP) to allow the passage of the oncoming ant (WALK). The time loss per collision was measured and it did not differ according to the type of ants involved. When an ant gave way to another, it generally moved to the side of the trail and allowed the passage of the ant. The follower ants might benefit from the passage of the leading ant (the ant that was given way) before the ant that gave way returns to the top of the trail. This latter effect corresponds to a cooperative behavior between ants because the subsequent ants benefit from the passage of the leading ant. The probability of an ant to benefit from the passage of the leading ant depended both on its position as a follower and on the category of the leading ant (O, U or L). N indicates the number of observation used to obtain the mean value or rule. (DOCX) [file pcbi.1006523.s001.docx]

| Levels | Measure | Results |
| --- | --- | --- |
| Foraging efficiency | Proportion of Laden ants leaving the food source | **Mean=0.24** Min=0.19 Max=0.28 |
| Collective level | Total number of ants  Temporal Traffic organization | **Mean=5418** Min=2819 Max=6317 (N=12)  **Mean cluster size=5.2** Min=1 Max=73 (N=5242) |
| Individual level | Speed  Priority rules  Time loss per collision  Cooperative behavior | O: 2.38 cm.s-1 (N=110)  U: 2.28 cm.s-1 (N=110)  L: 1.91 cm.s-1 (N=110)  U(L): 1.84 cm.s-1 (N=110)  O vs L => O stops, L walks (N=300)  O vs U => U stops, O walks (N=400)  U vs L => U stops, L walks (N=110)  0.8 s (N=100)  L leading => benefit 1 to 15 U (N=300) |
