# Supplementary material for: Self-organized traffic via priority rules in leaf-cutting ants
Source: PLoS Comput Biol. 2018 Oct 11;14(10):e1006523. doi: 10.1371/journal.pcbi.1006523 (PMC6198993; doi:10.1371/journal.pcbi.1006523)
Supplement: S2 Table — From [20] based on 12 replicates. The trail linking the nest to the food source was 5cm wide and 300cm long. The flow of ants leaving the nest (outbound ants) and that leaving the food source (inbound ants) was counted in 1 min intervals for 1h and the proportion of laden ants in the inbound flow was measured. The formation of groups of successive ants travelling in the same direction in the sequence of ants observed on the trail was quantified. At the individual level, the ants speed and the outcome of head-on collisions between ants (priority rule) were measured. Three types of ants were distinguished: outbound ants (O), unladen (U) ants and laden ants (L). The outcome of head-on collisions between outbound ants and inbound laden ants (O vs L), and outbound ants and inbound unladen ants (O vs U) were analyzed. Typically, after a collision occurred one ant turns (TURN) to allow the passage of the oncoming ant (WALK). N indicates the number of observation used to obtain the mean value or rule. (DOCX) [file pcbi.1006523.s002.docx]

| Levels | Measure | Results |
| --- | --- | --- |
| Foraging efficiency | Proportion of Laden ants leaving the food source | **Mean=0.04** Min=0.02 Max=0.06 |
| Collective level | Total number of ants  Temporal Traffic organization  Spatial traffic organization | **Mean=8803** Min=6659 Max=11498 (N=12)  **Mean cluster size =1.9** Min=1 Max=14 (N=20655)  O: 0.64 ants => central zone  U: 0.39 ants => central zone  L: 0.91 ants => central zone |
| Individual level | Speed  Priority rules  Time loss per collision | O: 2.43 cm.s-1 (N=100)  U: 2.26 cm.s-1 (N=100)  L: 2.11 cm.s-1 (N=50)  O vs L => O turns, L walks (N=160)  O vs U => U turns, O walks (N=540)  0.8 s (N=100) |
