# Supplementary figures and images for: Self-organized traffic via priority rules in leaf-cutting ants
Source: PLoS Comput Biol. 2018 Oct 11;14(10):e1006523. doi: 10.1371/journal.pcbi.1006523 (PMC6198993; doi:10.1371/journal.pcbi.1006523)

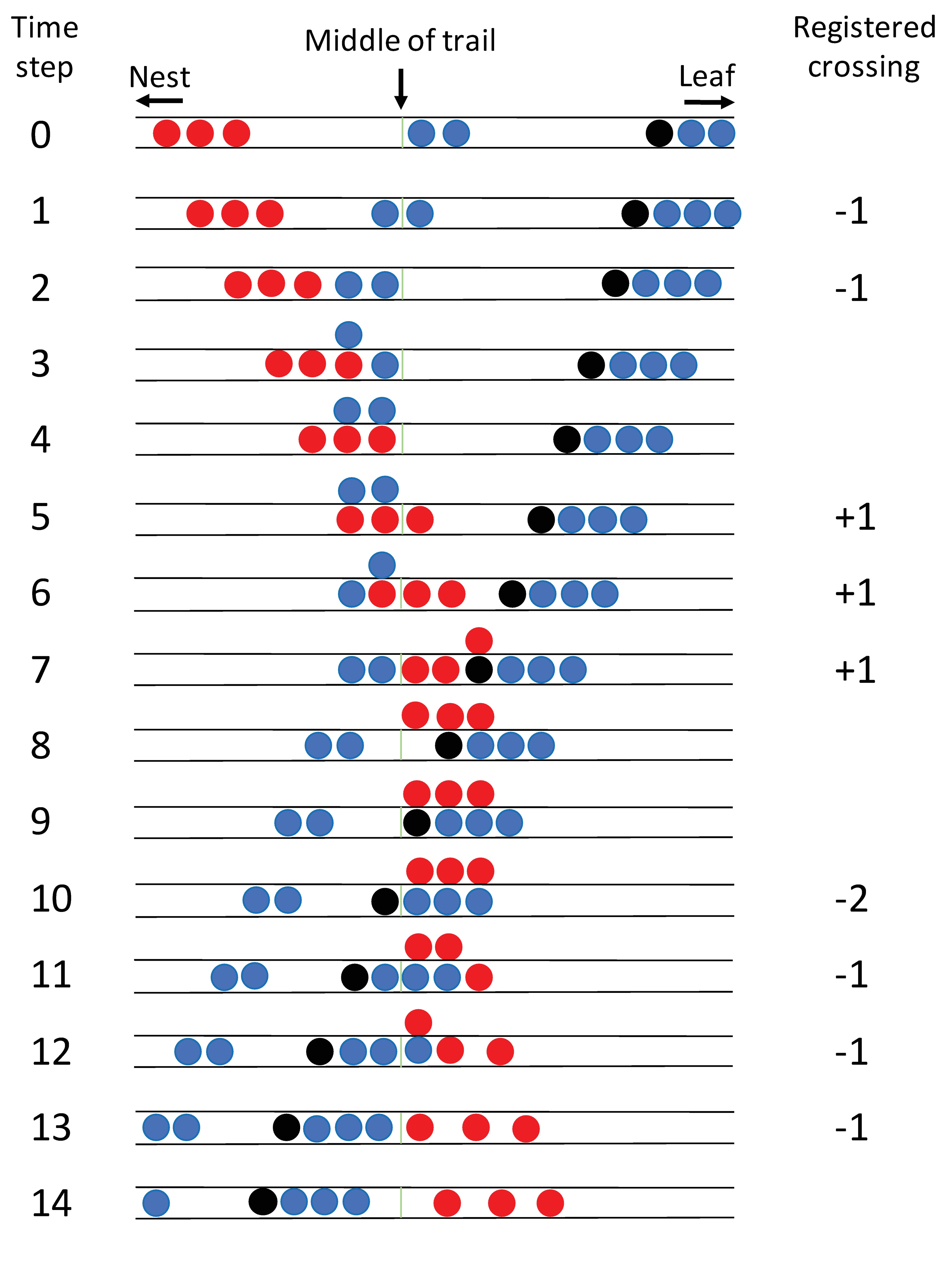

Supplement: S1 Fig — At time 0 there are outbound particles (red dots), inbound unladen particles (blue dots), and an inbound laden particle (black dot) near the middle of the trail. Over time the red particles will move towards the leaf source (right) and the black and blue particles towards the nest (left), and when a particle crosses the middle of the trail we record a crossing as +1 if it is outbound, -1 if inbound unladen, and -2 if inbound laden. We note that on time steps 1 and 2 we record inbound crossings (-1). On time step 3 the first interaction where one unladen particle steps off the trail in order to give way to an outbound ant. This process continues according to the model rules and by the time step 14 we have collected the sequence -1,-1,+1,+1,+1,-2,-1,-1,-1 and from this we calculate group size by counting consecutive entries with the same sign. Here we first had a group of 2 inbound particles (-1,-1), then a group of 3 outbound particles (+1,+1,+1), and then a group of 4 inbound particles (-2,-1,-1,-1). This phenomenon is referred to as a de-synchronization of inbound and outbound traffic involving the formation of alternating groups of inbound and outbound ants in [22] and we use the same terminology for particles here. (TIF) [file pcbi.1006523.s003.tif]
